# Apoptosis of the fibrocytes type 1 in the spiral ligament and blood labyrinth barrier disturbance cause hearing impairment in murine cerebral malaria

**DOI:** 10.1186/1475-2875-11-30

**Published:** 2012-02-01

**Authors:** Joachim Schmutzhard, Christian H Kositz, Rudolf Glueckert, Erich Schmutzhard, Annelies Schrott-Fischer, Peter Lackner

**Affiliations:** 1 Department of Otorhinolaryngology, Innsbruck Medical University, Anichstraβe 35, 6020 Innsbruck, Austria; 2 Department of Neurology, Innsbruck Medical University, Anichstraβe 35, 6020 Innsbruck, Austria

**Keywords:** Murine cerebral malaria, Hearing impairment, Apoptosis, Spiral ligament fibrocytes, Blood labyrinth barrier

## Abstract

**Background:**

Experimental murine malaria has been shown to result in significant hearing impairment. Microscopic evaluation of the temporal bones of these animals has revealed regular morphology of the cochlea duct. Furthermore, the known vascular pathologic changes being associated with malaria could not be found. Immunohistochemistry for ICAM1 showed a strong marking in the *stria vascularis*, indicating a disturbance of the endocochlear potential. The aim of this study was to evaluate the role of apoptosis and the disturbance of the blood labyrinth barrier in the murine malaria associated hearing impairment.

**Methods:**

The temporal bones of seven mice with cerebral malaria-four with hearing impairment, three without hearing impairment-were evaluated with immunohistochemistry for cleaved caspase 3 to detect apoptosis and connexin 26, a gap junction protein being a cornerstone in the endocochlear potassium recirculation. Furthermore five animals with cerebral malaria were treated with Evans blue prior to sacrification to detect disturbances of the blood labyrinth barrier.

**Results:**

Cleaved caspase 3 could clearly be detected by immunohistochemistry in the fibrocytes of the spiral ligament, more intensively in animals with hearing impairment, less intensively in those without. Apoptosis signal was equally distributed in the spiral ligament as was the connexin 26 gap junction protein. The Evans blue testing revealed a strong signal in the malaria animals and no signal in the healthy control animals.

**Conclusion:**

Malfunction of the fibrocytes type 1 in the spiral ligament and disruption of the blood labyrinth barrier, resulting in a breakdown of the endocochlear potential, are major causes for hearing impairment in murine cerebral malaria.

## Background

Malaria still is one of the most frequent infectious diseases worldwide. Especially, *Plasmodium falciparum *malaria is known for its severe course with multi-organ involvement [1]. Severe neurocognitive and language developmental impairment in sub-Saharan children following a severe course of malaria have been reported [2,3]. Hearing impairment during childhood is known to be one of the fundamental causes of acquired language disorders. A possible association between fever and hearing impairment has been corroborated by the fact that a high percentage of deaf Tanzanian children had a positive medical history of severe fever-mostly due to malaria or pneumonia [4]. Out of 23 children with neurocognitive impairment after cerebral malaria, 14 suffered from a loss of speech and nine from hearing impairment [5]. This clinical data point towards an involvement of the inner ear in cerebral malaria.

Recent work by Schmutzhard *et al. *did show a significant hearing impairment in mice with cerebral malaria [6]. The light-microscopic examination of the affected temporal bones did not show any direct alteration of the cochlea, such as loss of outer haircells. Furthermore, the malaria typic vascular alterations, such as leukocyte sequestration and microhaemorrhages, could not be detected. The only interesting pathologic change described so far in the malaria-impaired cochlea was an up-regulation of ICAM 1 in the *stria vascularis *[7]. Thus, so far the pathologic alterations leading to the observed hearing impairment could not be objectivized. The ICAM 1 up-regulation in the *stria vascularis *suggests an alteration of the endocochlear potential.

The spiral ligament of the cochlea duct is known to play an essential role in the ionic composition of the endolymph. This task is accomplished by a complex connection of various gap junctions between the supporting cells of the organ of Corti and the different fibrocytes of the spiral ligament [8]. These gap junctions in the lateral wall of the cochlea have been shown to contain various connexins in different regions of the cochlea, like connexin 26, 30, 31 and 43 [9]. Connexin 26 mutations are known to be a major cause for non-syndromic deafness [10].

Up-regulated proinflammatory cytokines, as seen in murine cerebral malaria [11], are known to result in a damage of cultured fibrocytes of the spiral ligament [12]. In the inner ear apoptosis is a frequent reaction to various pathological stimuli [13], e.g. an activation of cleaved caspase 3 has been shown in an animal model for Ménière's disease in the stria vascularis, the fibrocytes of the spiral ligament and the organ of Corti [14]. Various infectious diseases, such as pneumococcal meningitis, have been shown to affect the blood labyrinth barrier [15]. The proper configuration of the blood labyrinth barrier, which is also maintained by the fibrocytes of the spiral ligament, is essential for the electrolyte circulation in the inner ear. Such a disturbance of this circulation leads to a disruption of the endocochlear potential [16].

On the one hand, the aim of this study is to evaluate the affection of the blood labyrinth barrier. On the other hand, the role of apoptosis in malaria-affected temporal bones is examined and the findings are correlated with the gap junction system in the lateral wall. A cellular pathologic alteration in these parts of the cochlea would explain the audiologic malfunction in combination with the morphologic integrity of the cochlea as previously described [7].

## Methods

The animal studies conformed to the Austrian guidelines for the care and use of laboratory animals and were approved by the Austrian Ministry for Education, Science and Culture with the reference number do. Zl. A08/4102.

### Study design

Temporal bones of seven mice with cerebral malaria and three control mice were evaluated. As previously published, four of the included cerebral malaria mice showed hearing impairment of 20 dB or more in one or more frequencies. The histologic evaluation had not revealed any morphologic alteration, but an up-regulation of ICAM-1 in the *stria vascularis *of the cerebral malaria temporal bones [7]. These previously examined temporal bones were further evaluated with immunohistochemistry for cleaved caspase 3 and connexin 26. The protocol of the ABR measurements and the specimen preparation of the included temporal bones have been published previously [7].

Six mice were newly added to the study, five of them infected with *Plasmodium berghei *according to the previously published malaria mouse model. The disease was closely monitored with the SHIRPA score and daily blood smears as published previously [7]. Animals developing cerebral malaria were injected with Evans blue to evaluate the blood labyrinth barrier.

### Animal model

The C57BL/6 mouse is a strain susceptible to blood stages of *Plasmodium berghei *ANKA (PbA). After the determination of the aABR thresholds as previously published [6], the mice are infected with an intraperitoneal application of 5 × 10^6 ^parasitized erythrocytes of a homologue donor, which had been infected with frozen polyclonal stocks of PbA [17,18]. With daily blood smears of tail blood the parasitaemia is monitored. The smears are stained with Giemsa.

The progress of the disease is evaluated with the SHIPRA score as previously described [19]. When typical signs and symptoms of cerebral malaria, like convulsions, paralysis and coma appear, the mice are deeply anaesthesized, sacrificed and the temporal bones and brains removed for further histomorphologic evaluation [7].

### Immunohistochemistry

Cleaved caspase 3 (Asp175) (Cell Signalling Technology, Danvers, MA, USA) was used to detect apoptosis in the cochlea. Connexin 26 was visualized with a polyclonal rabbit anti-cx26 (Invitrogen, Carlsbad, CA, USA). The immunohistochemical staining was done with a Ventana^® ^Roche^® ^Discovery Immunostainer according to the DAB-MAP discovery research standard procedure. Counterstaining was done with Hematoxylin II (Ventana^® ^Roche^® ^).

Three slides with five different sections of the cochlea were evaluated in a double-blinded manner by two independent ENT specialists (JS, ASF) in a semi-quantitative manner: ++ strong reaction, + mild reaction, - no reaction. Additionally, the area of the spiral ligament in the middle turn was measured with AxioVs40 V 4.8.1.0 (Carl Zeiss). The positively staining fibrocytes were counted and correlated with the measured area of the spiral ligament.

Digital images of the sections were acquired using an Image-pro^® ^6.0 analysis system (Media Cybernetics^®^, Silver Spring, USA) linked to a 3 CCD colour video camera (Sony DXC-950P) on an Olympus BX 50 light microscope.

### Evans blue

The animals with cerebral malaria, verified by a pathologic SHIRPA score and positive blood smears, were injected with a 1% solution of Evans blue 1 h prior to scarification. After preparation of the cochlea the oval and the round window were perforated. The submersion fixation was done with 4% paraformaldehyde for 48 h. The decalcification of the temporal bones was done with 1% ascorbic acid, 0,8% sodium chloride solved in aqua. The pH-value was kept between 2.5 and 2.6. After 24 h the decalcification, which was supported by an electrolytic bone decalcifier (MEDAX 33000^®^, Neumünster, Germany), was completed. All specimens were cryo-embedded according to the protocol as described by Coleman *et al. *[20]. Serial sections (10 μm) were cut on a cryostat (LEICA CM 3050), mounted on glass slides (SuperFrost^® ^Plus, Menzel-Gläser, Braunschweig, Germany), air-dried for 1 h and stored at -20°C for use in further work.

The brains were fixed with submersion in 4% paraformaldehyde for macroscopic evaluation. The fluorescence microscopic evaluation was done with a Carl Zeiss Axio Imager M1 with a plan-apochromat 20×/0.60 lens and a shutter speed of 230-235 ms.

## Results

### Animal experiments

**Figure 1:**
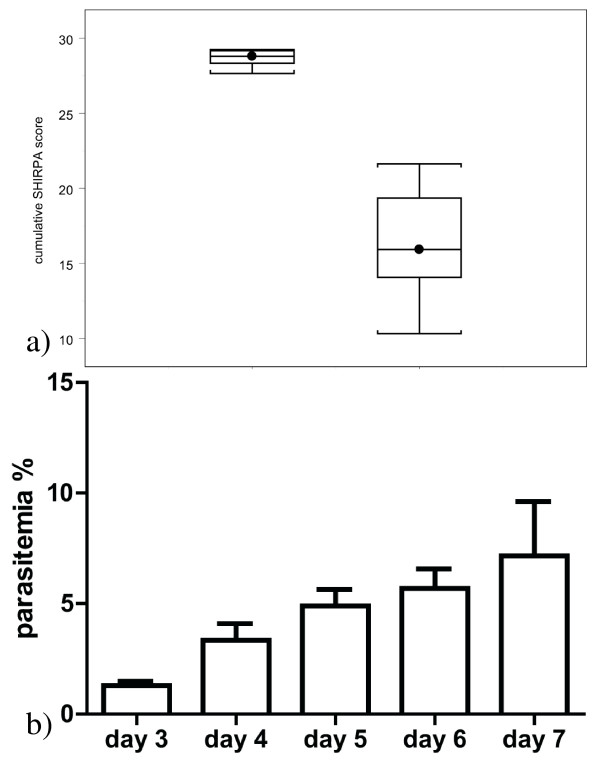
**demonstrates the course of the disease**. a) In the left box plot the SHIRPA score before infection is demonstrated. The right box plot shows the SHIRPA scores at the time point of sacrification. b) demonstrates the parasitaemia evaluated on day three to seven.

The established animal model did show the expected course. The included animals did develop cerebral malaria between days 5 and 7 after infection (Table 1). On day 0 the SHIRPA-score ranged from 27.9 to 29.5. At the time point of sacrification the SHIRPA ranged from 21.4 to 10.3 (Figure 1a) Over the course of the disease the parasitaemia gradually increased to 9.5% on day 7 (Figure 1b)

**Table 1:** lists the immunohistochemical results for cleaved Caspase 3 and the hearing ability of the animals

Animal number	Malaria manifestation	**ED **[7]	Hearing loss	Cleaved Caspase 3					
				
				IHC	OHC	SGC	SV	SL	Li
CM1	cerebral	6	No	-	-	-	-	+	-

CM2	cerebral	7	Yes	-	-	+	-	++	-

CM3	cerebral	5	Yes	-	-	+	-	+	+

CM4	cerebral	6	Yes	-	-	+	-	++	+

CM5	cerebral	5	Yes	-	-	+	-	++	+

CM6	cerebral	7	No	-	-	-	-	+	-

CM7	cerebral	7	No	-	-	-	-	+	-

CO1	not infected	11	No	-	-	-	-	-	-

CO2	not infected	11	No	-	-	-	-	-	-

CO3	not infected	11	No	-	-	-	-	-	-

The evaluation of the slices was done in a semi-quantitative manner: ++ strong reaction, + mild reaction,-no reaction. IHC: inner hair cells, OHC: outer hair cells, SGC: spiral ganglion cells, SV: *stria vascularis*, SL: spiral ligament, Li: limbus, ED: euthanisia day after infection

### Cleaved caspase 3

**Figure 2:**
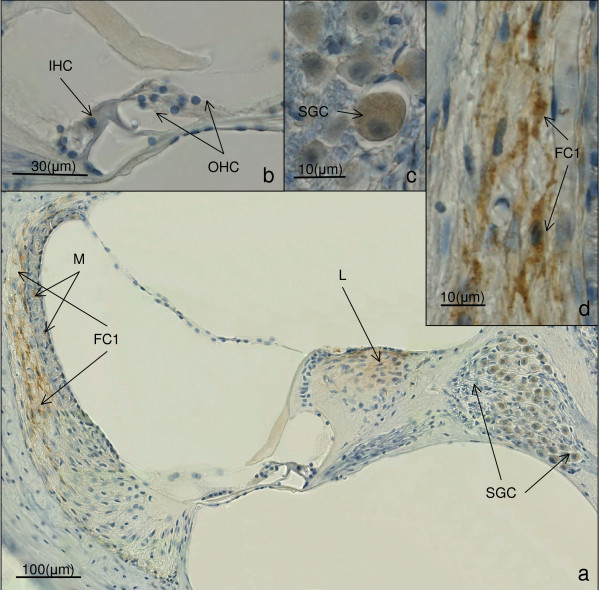
**shows the results of mouse CM 5-a hearing impaired animal-for cleaved caspase 3 immunohistochemistry**. A positive cleaved caspase 3 reaction is brown. a) shows an overview of the cochlea duct in the middle turn. The brown staining in the fibrocytes type 1 of the spiral ligament (FC1), the spiral ganglion cells (SGC) and the limbus (Li) indicates positive labelling for cleaved caspase 3. (magnification 200×). The brown staining in the stria vascularis is a physiologic melanin (M) pigmentation. b) shows the organ of Corti. The inner hair cells (IHC) and the outer hair cells (OHC) are well preserved. No immunohistochemical labelling can be visualized. c) shows a close up of the spiral ganglion cells (SGC) with a positive immunohistochemical labelling for cleaved caspase 3. d) visualizes the positive cleaved caspase 3 reaction in the fibrocytes type 1 of the spiral ligament.

The histologic slices of the included temporal bones showed well preserved structures. Immunohistochemistry for cleaved caspase 3 in the hearing impaired animals revealed a mild positive staining in the spiral ganglion neurons of all four hearing impaired temporal bones. Furthermore, a positive staining could be detected in the limbus of three of the four hearing impaired cochleae. The most intensive labelling could be seen in the fibrocytes of the spiral ligament neighbouring the *stria vascularis*, being even more intensive in the basal turns of the cochleae. The staining intensity decreased gradually in the middle and the upper coils. This pattern was observed in all four hearing impaired temporal bones. No apoptotic activity could be found in the *stria vascularis*, the outer and the inner haircells (Figure 2a-d, Table 1).

**Figure 3:**
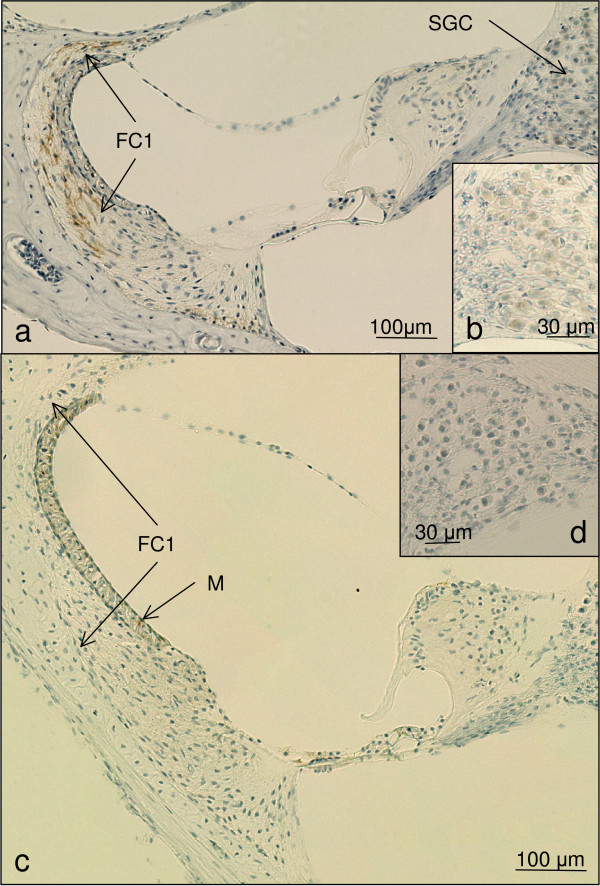
**a-b show the immunohistochemical result of the hearing animal CM 1 (magnification 200 ×)**. An immunohistochemical staining for cleaved caspase 3 can be detected in the fibrocytes type 1 (FC1) of the spiral ligament. No positive staining can be noted in the spiral ganglion cells (SGC). b) shows a close up of the non reacting spiral ganglion cells. c-d) show the immunohistochemical results for cleaved caspase 3 in the cochlea duct of the control animal CO1. No labelling could be detected in these structures (magnification 200 ×). No activation of cleaved Caspase 3 can be detected in these histological slices. The brown staining in the stria vascularis is a physiologic Melanin (M) pigmentation.

The immunohistochemistry for cleaved caspase 3 in the temporal bones of malaria mice without hearing impairment revealed a positive labelling in the fibrocytes of the spiral ligament in a similar pattern with less intensity as observed in the hearing impaired animals. No immunohistochemical alteration could be detected in the spiral ganglion neurons, the limbus, the *stria vascularis*, the outer and inner haircells (Figure 3a and 3b, Table 1).

The three control animals showed no immunolabelling in the fibrocytes of the spiral ligament, the spiral ganglion neurons, the *stria vascularis*, the limbus and the outer and inner haircells (Figure 3c and 3d, Table 1).

In the hearing impaired animals the evaluated area of the spiral ligament measured between 21,261 and 28,291 μm^2^, in mice without hearing impairment between 25,074 and 27,089 μm^2^. The cell count in animals with hearing impairment ranged from 17 to 23 positively labelling fibrocytes, in animals without hearing impairment from 5 to 11 cleaved Caspase 3 positive fibrocytes. In animals with hearing loss the cell/μm^2 ^ratio ranged between 0.00077762 and 0.000954, in animals without hearing loss from 0.00019239 to 0.00043869(Table 2).

**Table 2:** lists the results for the quantitative evaluation of the cleaved Caspase 3 positive fibrocytes in the spiral ligament.

Animal number	Hearing loss	Caspase 3 positive fibrocytes	Evaluated area(μm^2^)	Cells/μm^2 ^ratio
CM2	Yes	22	28291,5	0,00077762

CM3	Yes	17	21261,5	0,00079957

CM4	Yes	23	24109	0,00095400

CM5	Yes	21	25835,4	0,00081284

CM1	No	11	25074,5	0,00043869

CM6	No	6	27308,8	0,00021971

CM7	No	5	25988,3	0,00019239

The cell count is correlated to the evaluated area

### Connexin 26

**Figure 4:**
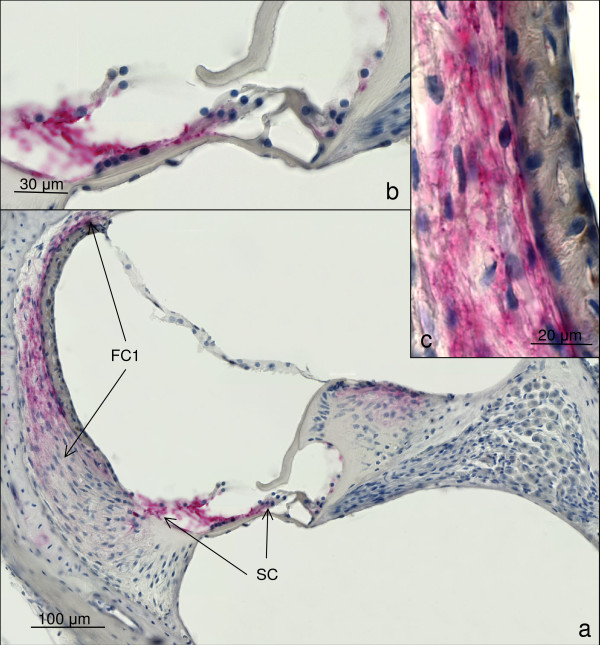
**shows the results for immunohistochemical detection of connexin 26**. A positive reaction is red. a) shows an overview of the cochlea duct at the middle turn of mouse CM5. A positive staining for connexion 26 can be detected in the upper part of the spiral ligament, consisting of fibrocytes type 1 (FC1) and the supporting cells of the organ of Corti (SC). (magnification 200×) b) shows a close up of the organ of Corti. The red colour denotes the positive area for connexin 26. (magnification 1,000×) c) is a close up view of the *stria vascularis *and the spiral ligament in the region of the fibrocytes type 1. The red colour represents the connexin 26 distribution in the upper part of the spiral ligament. (magnification 1,000×).

In all temporal bones a positive immunolabelling for Connexin 26 could be detected in the fibrocytes of the spiral ligament neighbouring the *stria vascularis*. Furthermore, a positive staining could be detected in the limbus and the supporting cells of the organ of Corti, such as the Claudius cells and the Deiter cells. No positive staining could be detected in the *stria vascularis*, the spiral ganglion neurons, the inner and outer hair cells (Figure 4a-c). The staining did not show any differences between the cerebral malaria animals and the control animals.

### Evans blue

**Figure 5:**
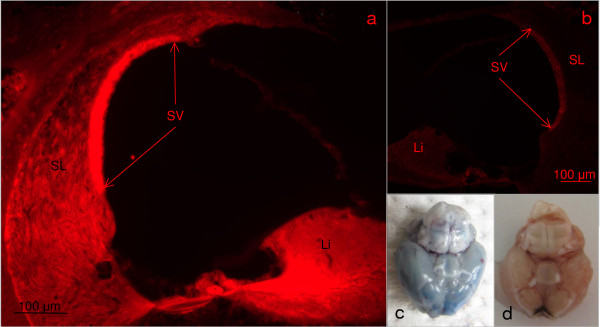
**shows the Evans blue evaluation**. a) demonstrates fluorescence microscopic evaluation of mouse Evans CM 4. The red colour signals extravasation of the Evans blue in the tissue, indicating a disturbance of the blood labyrinth barrier. A strong staining can be detected in the *stria vascularis *(SV) and the limbus (Li). Furthermore positive staining can be detected in the spiral ligament (SL). b) shows the fluorescence microscopic result for the control mouse Evans control. No extravasion of Evans blue can be detected. The shutter time with 231 ms is equal to Figure 4a. c) shows the gross morphology of the brain of the mouse Evans CM 4. The blue colour indicates a breakdown of the blood brain barrier. d) shows the gross morphology of the brain of the mouse Evans Control. No extravasation of Evans blue is visible, indicating a proper function of the blood brain barrier.

The fluorescence microscopic evaluation of the temporal bones of animals treated with Evans blue did show an intensive staining in the stria vascularis and the limbus. The spiral ligament showed a bright red colour (Figure 5a, Table 3). The spiral ganglion showed an intensive staining as well (Table 3). In the control animals, no specific red marking could be visualized (Figure 5b, Table 3).

**Table 3:** shows the semiquantitative evaluation of the Evans blue staining

Mouse	*Stria vascularis*	Limbus	Spiral ligament	Spiral ganglion
Evans CM 1	++	++	+	++

Evans CM 2	++	++	+	++

Evans CM 3	++	++	+	++

Evans CM 4	++	++	+	++

Evans CM 5	++	++	+	++

Evans Control	-	-	-	-

The evaluation of the slices was done in a semi-quantitative manner: ++ strong reaction, + mild reaction,--no reaction

The macroscopic evaluation of the brains showed a blue colour of the brain in all mice after Evans blue injection, the control animals a normal pink colouring (Figure 5c and 5d).

## Discussion

Immunohistochemistry in the hearing-impaired animals shows that cerebral malaria leads to an activation of organized cell death in various structures of the inner ear, mainly in the fibrocytes type 1 of the spiral ligament. Minor activation could be noted in the spiral ganglion neurons and the limbus of the hearing-impaired animals. The hearing animals showed a mild activation of the apoptotic cascade in the fibrocytes type 1 of the spiral ligament.

The immunohistochemical staining for connexin 26 showed an expected positive marking in the region of the fibrocytes type 1 and the supporting cells of the organ of Corti in both malarial and control animals. This expected staining pattern, described by Forge *et al.*, confirms the quality of the immunohistochemical experiment [9].

The localization in the spiral ligament of the cleaved Caspase 3 positive labelled fibrocytes is identical with the known region of the type 1 fibrocytes in the spiral ligament [21]. This pattern suggests that the fibrocytes type 1 are affected by the malaria-induced apoptosis. Fibrocytes type 1 play an essential role in the K^+ ^circulation of the cochlea [8]. A disruption of this functional unit as in the case of apoptosis of the fibrocytes type 1, would result in a depletion of the K^+ ^concentration in the endolymph resulting in the disturbance of the endocochlear potential. The fundamental role of these fibrocytes in maintaining the cochlear balance has been shown by Minowa *et al. *[16] who described in a mouse model for severe deafness no light-microscopic changes similar to the results of this study. The electron-microscopic evaluation however, revealed a remarkable alteration in the fibrocytes of the spiral ligament as the main cause of the functional impairment. A similar situation can be posted for the cerebral malaria mice.

The evaluation of the connexin 26 expression found in the hearing-impaired malaria animals, the hearing malaria animals and control animals did not reveal any alterations. Therefore, it can be postulated that malaria at this point of disease does not affect the connexin 26 expression itself. Nevertheless, it is safe to assume that activation of apoptosis in the fibrocytes will affect active duties, like potassium transportation. The examination of the expression pattern of the connexins 30, 31 and 43 in the cochlea would be essential to exclude a downregulation of these gap junction proteins as a fundamental cause of hearing loss in malaria. Further experiments will be necessary to clarify this point.

The previously published murine physiological data, showing a significant hearing impairment in the high frequencies (36 kHz), the low frequencies (8 kHz) and a moderate hearing impairment in the middle frequencies (13 kHz) partially supports these findings [6]. The light-microscopic evaluation of the hearing-impaired cochleae showed intensive immunohistochemical activity in the basal turn. Following the spiral ligament up the cochlea to the apical coil, the intensity of the immunostaining gradually fades. The weakest signal can be observed in the apical turn. This topographical distribution of the apoptosis activation correlates with the hearing impairment in the high frequency (36 kHz) and the middle frequencies (13 kHz), which are generated at the basal and middle turns of the cochlea. The cleaved Caspase 3 activation with a fading signal in the apical turn cannot explain the observed hearing loss in the low frequencies. This discrepancy suggests that additional pathophysiologic mechanisms, like breakdown of the blood labyrinth barrier may contribute to the hearing loss.

In malaria mice an up-regulation of cleaved Caspase 3 in the fibrocytes of the spiral ligament, the limbus and the spiral ganglion can be demonstrated. A similar activation pattern in the cochlea could be shown by Labbe *et al. *in an animal model of Ménière's disease. In this experiment, an activation of cleaved Caspase 3 in the fibrocytes of the spiral ligament, the stria vascularis and the spiral ganglion cells could be demonstrated, caused by oxidative stress leading to a hearing impairment. The equal pathologic alteration found in these two animal models suggests that oxidative stress may also play an essential role in the malaria-affected cochlea [14]. Recently, it has been shown that cerebral malaria induces apoptosis in brain endothelial cells, neurons and glia cells [22]. Wiese *et al. *related neuronal apoptosis with an increased immunoreactivity for 8-oxoguanine-a marker for oxidative stress-in the C57BL/6j mouse model [23]. A similar pathologic mechanism, as observed in the brain, is imaginable in the ear.

In the second part of this experiment, the function of the blood labyrinth barrier has been examined with intra-vital Evans blue injection. All five animals with cerebral malaria showed an intensive extravasation of Evans blue into the cochlear structures, whereas no staining could be found in the control animals. The macroscopic evaluation of the brains revealed a blue colouring of the diseased organs and no colouring of the control brains. The breakdown of the blood brain barrier during cerebral malaria is well known [24]. The strong fluorescence signal in the malaria animals proves a breakdown of the blood labyrinth barrier. The disturbance of the blood labyrinth barrier leads to a breakdown of the endocochlear potential, resulting in hearing loss [16]. In addition to this direct effect of the blood laryrinth barrier on the cochlea function, the protective role of the barrier is neutralized. Therefore, in murine malaria various inflammatory cytokines being up-regulated [11,25], their effect might be altered resulting in the pathologic alterations described above.

## Conclusion

Murine cerebral malaria leads to a disruption of the blood labyrinth barrier and to induction of apoptosis in fibrocytes of the spiral ligament. Both pathologic alterations for themselves and, even more, in combination result in a breakdown of the endocochlear potential. These results point to a breakdown of the endocochlear potential as the cause of the hearing loss observed in murine cerebral malaria.

## Competing interests

The authors declare that they have no competing interests.

## Authors' contributions

JS drafted the manuscript and evaluated the data. CK, RG made substantial contribution to the concept and the coordination of the animal experiments and the laboratory work. ASF evaluated the histologic data. ES contributed substantially to the design of the study and critically revised the manuscript. PL conceived and designed the study. All authors read and approved the final manuscript.
